# Supplementary material for: Genome-Wide Characterization of Soybean Hexokinase Genes Reveals a Positive Role of GmHXK15 in Alkali Stress Response
Source: Plants (Basel). 2023 Aug 30;12(17):3121. doi: 10.3390/plants12173121 (PMC10490225; doi:10.3390/plants12173121)
Supplement: Supplementary file 1 [file plants-12-03121-s001.zip › plants-2503916-supplementary.pdf]

Supplementary Materials

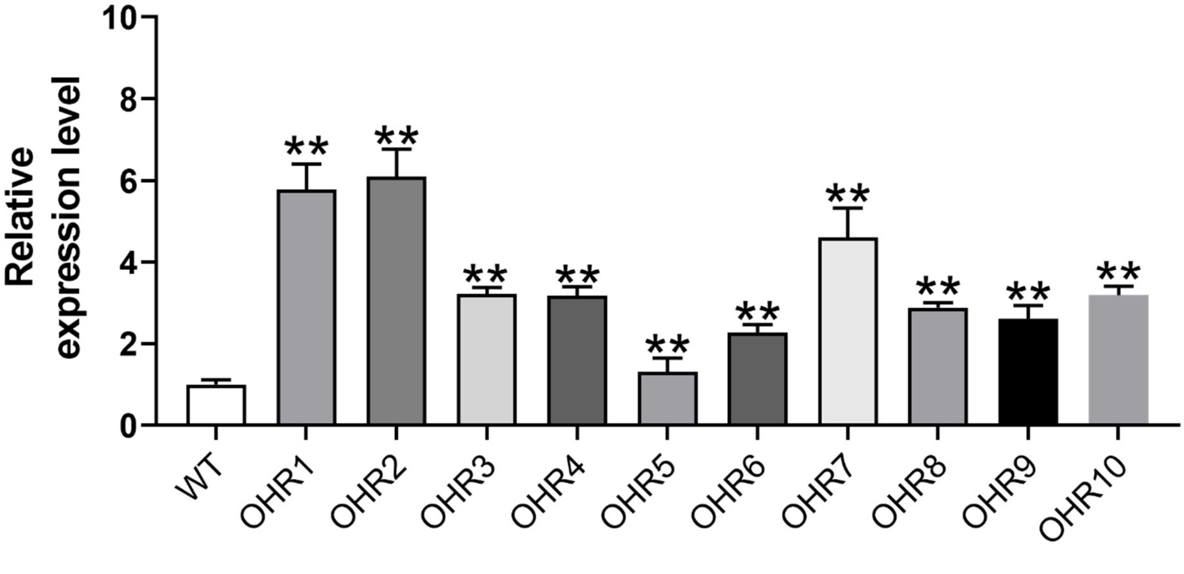

Figure S1. Identification of transgene expression in *GmHXK15* transgenic soybean hairy roots. The expression levels of *GmHXK15* in *GmHXK15*-overexpressing hairy roots (OHR) compared with the control hairy roots (CHR). Statistically significant differences from the control group, as determined by Student's t-test, are indicated by asterisks above the bars (** $p < 0.01$).

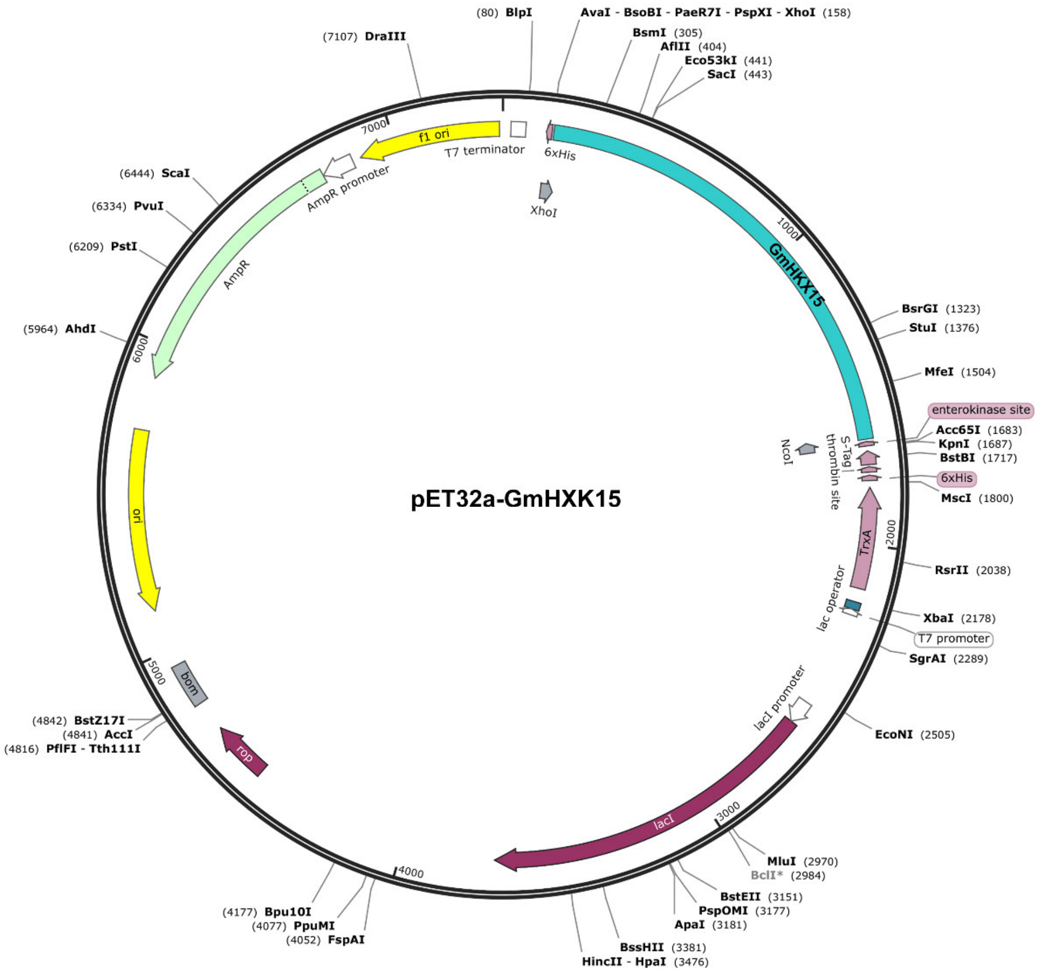

Figure S2. Diagram of constructing prokaryotic expression vector (pET32a-GmHXK15)

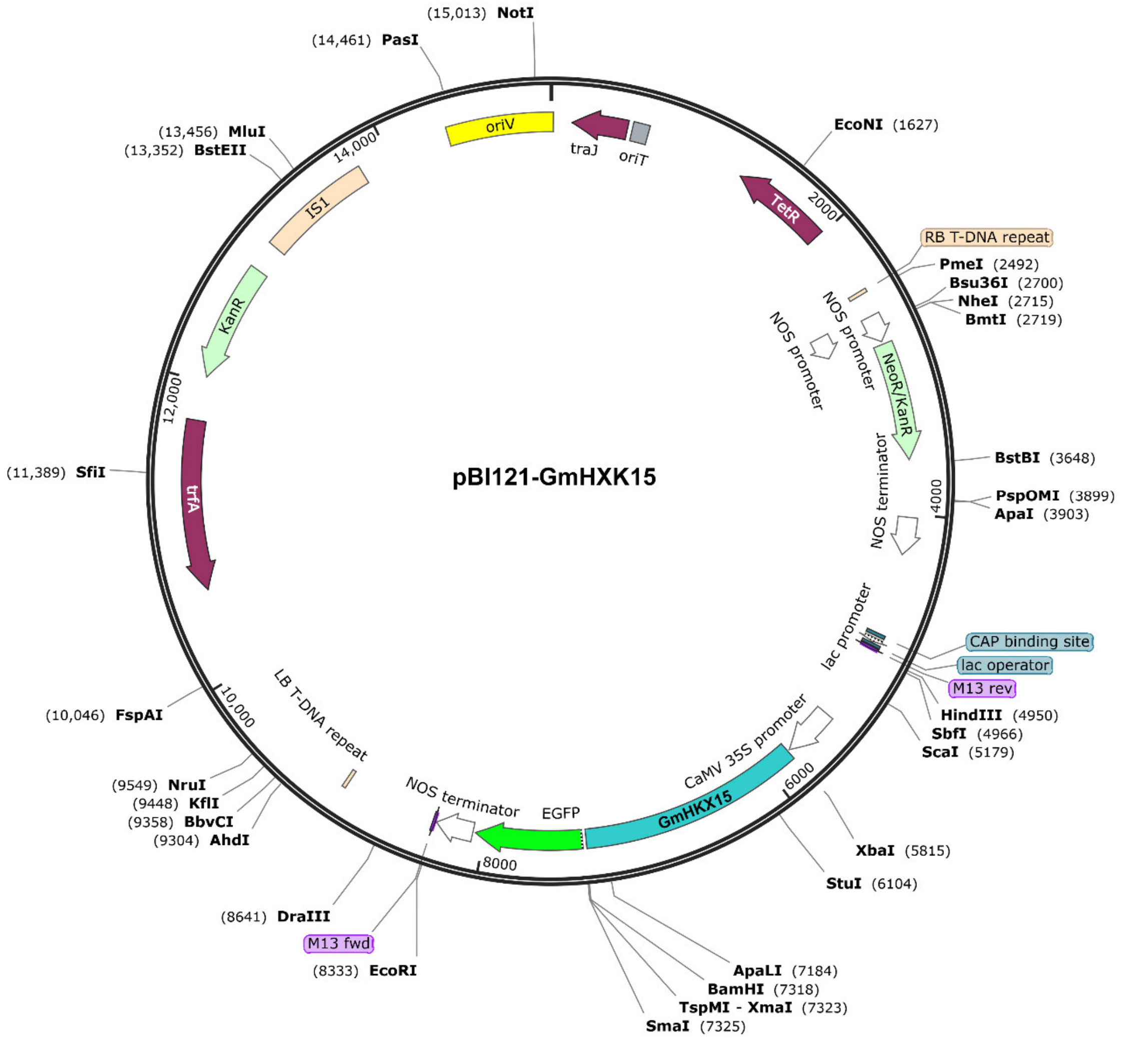

Figure S3.·Diagram of *GmHXK15* genes and the *eGFP* gene fusion.

Table S1. Basic information of *HXK* genes from *Arabidopsis* and soybean.

Gene Name	Gene ID	Gene location	ORF length (bp)	Protein length (aa)	Isoelectric point	Molecular weight (KDa)
AtHXK1	AT4G29130.1	Chr4: 14352036-14355103	840	280	8.90	31.07
AtHXK2	AT2G19860.1	Chr2: 8570749-8574000	1509	503	5.73	54.49
AtHXK3	AT1G47840.1	Chr1: 17616106- 17618894	1482	494	6.35	53.88
AtHXL1	AT1G50460.1	Chr1: 18693683-18697659	1497	499	5.55	54.60
AtHXL2	AT3G20040.1	Chr3: 6994893-6998171	1503	501	8.12	54.96
AtHXL3	AT4G37840.1	Chr4: 17790146-17792198	1482	494	5.72	54.24
GmHXK1	Glyma.17G182400.1	Chr17: 22439700-22440759	471	156	5.18	17.42
GmHXK2	Glyma.09G144600.1	Chr09: 35879525-35881559	891	296	6.22	32.92
GmHXK3	Glyma.17G257800.1	Chr17: 41189740-41193540	1503	500	5.48	53.39
GmHXK4	Glyma.14G218800.1	Chr14: 48395237-48399660	1506	501	5.11	53.73
GmHXK5	Glyma.05G110500.1	Chr05: 29291407-29297769	1473	490	6.26	53.66
GmHXK6	Glyma.17G156200.1	Chr17: 13283809-13288483	1473	490	6.30	53.85
GmHXK7	Glyma.01G226900.1	Chr01: 55523406-55528476	1497	498	8.76	54.39
GmHXK8	Glyma.11G015800.1	Chr11: 1104412-1109421	1497	498	8.60	54.59
GmHXK9	Glyma.11G095600.1	Chr11: 7249879-7254364	1515	504	6.66	55.04
GmHXK10	Glyma.12G021700.1	Chr12: 1566156-1570771	1515	504	6.34	54.80
GmHXK11	Glyma.07G015100.1	Chr07: 1184278-1191497	1218	405	5.66	44.26
GmHXK12	Glyma.05G226600.1	Chr05: 40495688-40500542	1497	498	5.58	53.70
GmHXK13	Glyma.01G007300.1	Chr01: 736360-740960	1491	496	6.56	53.85
GmHXK14	Glyma.08G200600.1	Chr08: 16223425-16229564	1479	492	5.54	53.66
GmHXK15	Glyma.08G033300.1	Chr08: 2667396-2672267	1497	498	5.65	53.61
GmHXK16	Glyma.01G007200.1	Chr01: 730041-735381	1491	496	5.96	53.65
GmHXK17	Glyma.07G124500.1	Chr07: 14792320-14800870	1497	498	5.95	53.64

Table S2. The gene ID and location of *HXK* genes used in this study.

Gene Name	Gene ID	Gene Location		
		Chromosome	Start	End
GmHXK1	Glyma.17G182400.1	Gm17	22439700	22440759
GmHXK2	Glyma.09G144600.1	Gm9	35879525	35881559
GmHXK3	Glyma.17G257800.1	Gm17	41189740	41193540
GmHXK4	Glyma.14G218800.1	Gm14	48395237	48399660
GmHXK5	Glyma.05G110500.1	Gm5	29291407	29297769
GmHXK6	Glyma.17G156200.1	Gm17	13283809	13288483
GmHXK7	Glyma.01G226900.1	Gm1	55523406	55528476
GmHXK8	Glyma.11G015800.1	Gm11	1104412	1109421
GmHXK9	Glyma.11G095600.1	Gm11	7249879	7254364
GmHXK10	Glyma.12G021700.1	Gm7	1184278	1191497
GmHXK11	Glyma.07G015100.1	Gm14	48395237	48399660
GmHXK12	Glyma.05G226600.1	Gm12	1566156	1570771
GmHXK13	Glyma.01G007300.1	Gm1	736360	740960
GmHXK14	Glyma.08G200600.1	Gm8	16223425	16229564
GmHXK15	Glyma.08G033300.1	Gm8	2667396	2672267
GmHXK16	Glyma.01G007200.1	Gm1	730041	735381
GmHXK17	Glyma.07G124500.1	Gm7	14792320	14800870
SbHXK1	Sobic.009G203500.1	Sb9	55223485	55229214
SbHXK2	Sobic.009G069800.1	Sb9	8119731	8123399
SbHXK3	Sobic.003G035500.1	Sb3	3255365	3259378
SbHXK4	Sobic.003G421201.1	Sb3	72624828	72631511
SbHXK5	Sobic.009G119100.1	Sb9	46791810	46796431
SbHXK6	Sobic.003G291800.1	Sb3	62430806	62437380
SbHXK7	Sobic.003G280400.1	Sb3	61559065	61563179
OsHXK1	LOC_Os07g26540.1	Os7	15292763	15294856
OsHXK2	LOC_Os05g45590.1	Os5	26418671	26422551
OsHXK3	LOC_Os01g71320.1	Os1	41305712	41314573
OsHXK4	LOC_Os07g09890.1	Os7	5256325	5259781
OsHXK5	LOC_Os05g44760.1	Os5	26017283	26022343
OsHXK6	LOC_Os01g53930.1	Os1	31009687	31013954
OsHXK7	LOC_Os05g09500.1	Os5	5337212	5341257
OsHXK8	LOC_Os01g09460.1	Os1	4820124	4823320
OsHXK9	LOC_Os01g52450.1	Os1	30131324	30135227
OsHXK10	LOC_Os05g31110.1	Os5	18075010	18081327
MtHXK1	Medtr5g009000.1	Mt5	2038574	2044069
MtHXK2	Medtr6g088795.1	Mt6	33918506	33923707
MtHXK3	Medtr8g102460.1	Mt8	43122065	43126983
MtHXK4	Medtr8g014530.1	Mt8	4574213	4578862
MtHXK5	Medtr1g025140.1	Mt1	8014049	8017043
PvHXK1	Phvul.002G308400.1	Pv2	47615533	47622160
PvHXK2	Phvul.004G175500.1	Pv4	47830230	47834331
PvHXK3	Phvul.011G023700.1	Pv11	2110284	2115063
PvHXK4	Phvul.010G144900.1	Pv10	42638980	42644829
PvHXK5	Phvul.002G034000.2	Pv2	3391386	3395957
PvHXK6	Phvul.003G231300.1	Pv3	46377921	46381916
PvHXK7	Phvul.001G007000.1	Pv1	450223	453962
AtHXK1	AT4G29130.1	At4	14352036	14355103
AtHXL1	AT1G50460.1	At5	18693683	18697659

AtHXK2	AT2G19860.1	At2	8570749	8574000
AtHXK3	AT1G47840.1	At1	17616106	17618894
AtHXL2	AT3G20040.1	At3	6994893	6998171
AtHXL3	AT4G37840.1	At4	17790146	17792198

Table S3. The syntenic relationships among *HXK* genes from *G. max*, *A. thaliana*, *O. sativa*, *P. vulgaris*, *M. truncatula* and *S. bicolor*.

Block	Glycine max gene location			Glycine max gene ID	Glycine max Gene name	Glycine max gene location			Glycine max Gene ID	Glycine max Gene name
	Chr	start	end			Chr	start	end		
49	Gm7	14792320	14800870	Glyma.07G124500.1	GmHXK17	Gm1	730041	735381	Glyma.01G007200.1	GmHXK16
566	Gm7	14792320	14800870	Glyma.07G124500.1	GmHXK17	Gm5	40495688	40500542	Glyma.05G226600.1	GmHXK12
788	Gm7	14792320	14800870	Glyma.07G124500.1	GmHXK17	Gm7	1184278	1191497	Glyma.07G015100.1	GmHXK11
872	Gm7	14792320	14800870	Glyma.07G124500.1	GmHXK17	Gm17	13283809	13288483	Glyma.17G156200.1	GmHXK6
806	Gm7	14792320	14800870	Glyma.07G124500.1	GmHXK17	Gm8	16223425	16229564	Glyma.08G200600.1	GmHXK14
801	Gm7	14792320	14800870	Glyma.07G124500.1	GmHXK17	Gm8	2667396	2672267	Glyma.08G033300.1	GmHXK15
629	Gm5	29291407	29297769	Glyma.05G110500.1	GmHXK5	Gm17	13283809	13288483	Glyma.17G156200.1	GmHXK6
1168	Gm11	1104412	1109421	Glyma.11G015800.1	GmHXK8	Gm11	7249879	7254364	Glyma.11G095600.1	GmHXK9
1175	Gm11	1104412	1109421	Glyma.11G015800.1	GmHXK8	Gm12	1566156	1570771	Glyma.12G021700.1	GmHXK10
86	Gm11	1104412	1109421	Glyma.11G015800.1	GmHXK8	Gm1	55523406	55528476	Glyma.01G226900.1	GmHXK7
898	Gm8	16223425	16229564	Glyma.08G200600.1	GmHXK14	Gm8	2667396	2672267	Glyma.08G033300.1	GmHXK15
59	Gm8	16223425	16229564	Glyma.08G200600.1	GmHXK14	Gm1	730041	735381	Glyma.01G007200.1	GmHXK16
574	Gm8	16223425	16229564	Glyma.08G200600.1	GmHXK14	Gm5	40495688	40500542	Glyma.05G226600.1	GmHXK12
790	Gm8	16223425	16229564	Glyma.08G200600.1	GmHXK14	Gm7	1184278	1191497	Glyma.07G015100.1	GmHXK11
571	Gm8	2667396	2672267	Glyma.08G033300.1	GmHXK15	Gm5	40495688	40500542	Glyma.05G226600.1	GmHXK12
795	Gm8	2667396	2672267	Glyma.08G033300.1	GmHXK15	Gm7	1184278	1191497	Glyma.07G015100.1	GmHXK11
1385	Gm17	41189740	41193540	Glyma.17G257800.1	GmHXK3	Gm14	48395237	48399660	Glyma.14G218800.1	GmHXK4
48	Gm7	1184278	1191497	Glyma.07G015100.1	GmHXK11	Gm1	730041	735381	Glyma.01G007200.1	GmHXK16
565	Gm7	1184278	1191497	Glyma.07G015100.1	GmHXK11	Gm5	40495688	40500542	Glyma.05G226600.1	GmHXK12
89	Gm11	7249879	7254364	Glyma.11G095600.1	GmHXK9	Gm1	55523406	55528476	Glyma.01G226900.1	GmHXK7
1174	Gm11	7249879	7254364	Glyma.11G095600.1	GmHXK9	Gm12	1566156	1570771	Glyma.12G021700.1	GmHXK10
1132	Gm5	40495688	40500542	Glyma.05G226600.1	GmHXK12	Gm1	730041	735381	Glyma.01G007200.1	GmHXK16
91	Gm12	1566156	1570771	Glyma.12G021700.1	GmHXK10	Gm1	55523406	55528476	Glyma.01G226900.1	GmHXK7
56	Gm8	2667396	2672267	Glyma.08G033300.1	GmHXK15	Gm1	730041	735381	Glyma.01G007200.1	GmHXK16
**Block**	**Glycine max gene location**			**Glycine max gene ID**	**Glycine max Gene name**	**Sorghum gene location**			**Sorghum bicolor Gene ID**	**Sorghum bicolor Gene name**
	**Chr**	**start**	**end**			**Chr**	**start**	**end**		
259	Gm7	14792320	14800870	Glyma.07G124500.1	GmHXK17	Sb3	3255365	3259378	Sobic.003G035500.1	SbHXK3
292	Gm8	16223425	16229564	Glyma.08G200600.1	GmHXK14	Sb9	55223485	55229214	Sobic.009G203500.1	SbHXK1
22	Gm1	730041	735381	Glyma.01G007200.1	GmHXK16	Sb9	8119731	8123399	Sobic.009G069800.1	SbHXK2

Block	Glycine max gene location			Glycine max gene ID	Glycine max Gene name	Medicago truncatula gene location			Medicago truncatula Gene ID	Medicago truncatula Gene name
	Chr	start	end			Chr	start	end		
408	Gm7	14792320	14800870	Glyma.07G124500.1	GmHXK17	Mt8	43122065	43126983	Medtr8g102460.1	MtHXK3
392	Gm7	14792320	14800870	Glyma.07G124500.1	GmHXK17	Mt6	33918506	33923707	Medtr6g088795.1	MtHXK2
627	Gm11	1104412	1109421	Glyma.11G015800.1	GmHXK8	Mt5	2038574	2044069	Medtr5g009000.1	MtHXK1
480	Gm8	16223425	16229564	Glyma.08G200600.1	GmHXK14	Mt8	4574213	4578862	Medtr8g014530.1	MtHXK4
478	Gm8	16223425	16229564	Glyma.08G200600.1	GmHXK14	Mt8	43122065	43126983	Medtr8g102460.1	MtHXK3
471	Gm8	2667396	2672267	Glyma.08G033300.1	GmHXK15	Mt8	43122065	43126983	Medtr8g102460.1	MtHXK3
460	Gm8	2667396	2672267	Glyma.08G033300.1	GmHXK15	Mt6	33918506	33923707	Medtr6g088795.1	MtHXK2
903	Gm17	41189740	41193540	Glyma.17G257800.1	GmHXK3	Mt1	8014049	8017043	Medtr1g025140.1	MtHXK5
407	Gm7	1184278	1191497	Glyma.07G015100.1	GmHXK11	Mt8	4574213	4578862	Medtr8g014530.1	MtHXK4
406	Gm7	1184278	1191497	Glyma.07G015100.1	GmHXK11	Mt8	43122065	43126983	Medtr8g102460.1	MtHXK3
776	Gm14	48395237	48399660	Glyma.14G218800.1	GmHXK4	Mt1	8014049	8017043	Medtr1g025140.1	MtHXK5
280	Gm5	40495688	40500542	Glyma.05G226600.1	GmHXK12	Mt8	43122065	43126983	Medtr8g102460.1	MtHXK3
278	Gm5	40495688	40500542	Glyma.05G226600.1	GmHXK12	Mt6	33918506	33923707	Medtr6g088795.1	MtHXK2
52	Gm1	730041	735381	Glyma.01G007200.1	GmHXK16	Mt8	43122065	43126983	Medtr8g102460.1	MtHXK3
41	Gm1	730041	735381	Glyma.01G007200.1	GmHXK16	Mt6	33918506	33923707	Medtr6g088795.1	MtHXK2
34	Gm1	55523406	55528476	Glyma.01G226900.1	GmHXK7	Mt5	2038574	2044069	Medtr5g009000.1	MtHXK1
**Block**	**Glycine max gene location**			**Glycine max gene ID**	**Glycine max Gene name**	**Phaseolus vulgaris gene location**			**Phaseolus vulgaris Gene ID**	**Phaseolus vulgaris Gene name**
	**Chr**	**start**	**end**			**Chr**	**start**	**end**		
469	Gm7	14792320	14800870	Glyma.07G124500.1	GmHXK17	Pv4	47830230	47834331	Phvul.004G175500.1	PvHXK2
449	Gm7	14792320	14800870	Glyma.07G124500.1	GmHXK17	Pv2	47615533	47622160	Phvul.002G308400.1	PvHXK1
321	Gm5	29291407	29297769	Glyma.05G110500.1	GmHXK5	Pv3	46377921	46381916	Phvul.003G231300.1	PvHXK6
765	Gm11	1104412	1109421	Glyma.11G015800.1	GmHXK8	Pv2	3391386	3395957	Phvul.002G034000.2	PvHXK5
1155	Gm17	13283809	13288483	Glyma.17G156200.1	GmHXK6	Pv3	46377921	46381916	Phvul.003G231300.1	PvHXK6
531	Gm8	16223425	16229564	Glyma.08G200600.1	GmHXK14	Pv2	47615533	47622160	Phvul.002G308400.1	PvHXK1
606	Gm8	16223425	16229564	Glyma.08G200600.1	GmHXK14	Pv10	42638980	42644829	Phvul.010G144900.1	PvHXK4
554	Gm8	2667396	2672267	Glyma.08G033300.1	GmHXK15	Pv4	47830230	47834331	Phvul.004G175500.1	PvHXK2
520	Gm8	2667396	2672267	Glyma.08G033300.1	GmHXK15	Pv2	47615533	47622160	Phvul.002G308400.1	PvHXK1
608	Gm8	2667396	2672267	Glyma.08G033300.1	GmHXK15	Pv10	42638980	42644829	Phvul.010G144900.1	PvHXK4
1145	Gm17	41189740	41193540	Glyma.17G257800.1	GmHXK3	Pv1	450223	453962	Phvul.001G007000.1	PvHXK7
505	Gm7	1184278	1191497	Glyma.07G015100.1	GmHXK11	Pv10	42638980	42644829	Phvul.010G144900.1	PvHXK4

448	Gm7	1184278	1191497	Glyma.07G015100.1	GmHXK11	Pv2	47615533	47622160	Phvul.002G308400.1	PvHXK1
982	Gm14	48395237	48399660	Glyma.14G218800.1	GmHXK4	Pv1	450223	453962	Phvul.001G007000.1	PvHXK7
804	Gm11	7249879	7254364	Glyma.11G095600.1	GmHXK9	Pv11	2110284	2115063	Phvul.011G023700.1	PvHXK3
348	Gm5	40495688	40500542	Glyma.05G226600.1	GmHXK12	Pv10	42638980	42644829	Phvul.010G144900.1	PvHXK4
329	Gm5	40495688	40500542	Glyma.05G226600.1	GmHXK12	Pv4	47830230	47834331	Phvul.004G175500.1	PvHXK2
297	Gm5	40495688	40500542	Glyma.05G226600.1	GmHXK12	Pv2	47615533	47622160	Phvul.002G308400.1	PvHXK1
843	Gm12	1566156	1570771	Glyma.12G021700.1	GmHXK10	Pv11	2110284	2115063	Phvul.011G023700.1	PvHXK3
44	Gm1	730041	735381	Glyma.01G007200.1	GmHXK16	Pv4	47830230	47834331	Phvul.004G175500.1	PvHXK2
9	Gm1	730041	735381	Glyma.01G007200.1	GmHXK16	Pv2	47615533	47622160	Phvul.002G308400.1	PvHXK1
11	Gm1	55523406	55528476	Glyma.01G226900.1	GmHXK7	Pv2	3391386	3395957	Phvul.002G034000.2	PvHXK5
**Block**	**Glycine max gene location**			**Glycine max gene ID**	**Glycine max Gene name**	**Arabidopsis thaliana gene location**			**Arabidopsis thaliana Gene ID**	**Arabidopsis thaliana Gene name**
	**Chr**	**start**	**end**			**Chr**	**start**	**end**		
1231	Gm1	55523406	55528476	Glyma.01G226900.1	GmHXK7	At4	14352036	14355103	AT4G29130.1	AtHXK1
571	Gm1	55523406	55528476	Glyma.01G226900.1	GmHXK7	At2	8570749	8574000	AT2G19860.1	AtHXK2
1188	Gm5	29291407	29297769	Glyma.05G110500.1	GmHXK5	At4	17790146	17792198	AT4G37840.1	AtHXL3
1383	Gm17	13283809	13288483	Glyma.17G156200.1	GmHXK6	At4	17790146	17792198	AT4G37840.1	AtHXL3
578	Gm8	16223425	16229564	Glyma.08G200600.1	GmHXK14	At2	8570749	8574000	AT2G19860.1	AtHXK2
573	Gm8	2667396	2672267	Glyma.08G033300.1	GmHXK15	At2	8570749	8574000	AT2G19860.1	AtHXK2
1251	Gm8	2667396	2672267	Glyma.08G033300.1	GmHXK15	At4	14352036	14355103	AT4G29130.1	AtHXK1
1199	Gm5	40495688	40500542	Glyma.05G226600.1	GmHXK12	At4	14352036	14355103	AT4G29130.1	AtHXK1
536	Gm5	40495688	40500542	Glyma.05G226600.1	GmHXK12	At2	8570749	8574000	AT2G19860.1	AtHXK2
1148	Gm1	730041	735381	Glyma.01G007200.1	GmHXK16	At4	14352036	14355103	AT4G29130.1	AtHXK1
473	Gm1	730041	735381	Glyma.01G007200.1	GmHXK16	At2	8570749	8574000	AT2G19860.1	AtHXK2
**Block**	**Sorghum bicolor gene location**			**Sorghum bicolor Gene ID**	**Sorghum bicolor Gene name**	**Sorghum bicolor gene location**			**Sorghum bicolor Gene ID**	**Sorghum bicolor Gene name**
	**Chr**	**start**	**end**			**Chr**	**start**	**end**		
132	Sb9	55223485	55229214	Sobic.009G203500.1	SbHXK1	Sb3	62430806	62437380	Sobic.003G291800.1	SbHXK6
133	Sb9	8119731	8123399	Sobic.009G069800.1	SbHXK2	Sb3	3255365	3259378	Sobic.003G035500.1	SbHXK3
126	Sb9	46791810	46796431	Sobic.009G119100.1	SbHXK5	Sb3	72624828	72631511	Sobic.003G421201.1	SbHXK4
99	Sb3	62430806	62437380	Sobic.003G291800.1	SbHXK6	Sb3	61559065	61563179	Sobic.003G280400.1	SbHXK7
**Block**	**Oryza sativa gene location**			**Oryza sativa Gene ID**	**Oryza sativa Gene name**	**Oryza sativa gene location**			**Oryza sativa Gene ID**	**Oryza sativa Gene name**
	**Chr**	**start**	**end**			**Chr**	**start**	**end**		

40	Os5	26418620	26422556	LOC_Os05g45590.1	OsHXK2	Os1	30131347	30135287	LOC_Os01g52450.1	OsHXK9
32	Os5	18075300	18081280	LOC_Os05g31110.1	OsHXK10	Os1	41305316	41314527	LOC_Os01g71320.1	OsHXK3
3	Os1	31009005	31014001	LOC_Os01g53930.1	OsHXK6	Os1	30131347	30135287	LOC_Os01g52450.1	OsHXK9
40	Os1	31009005	31014001	LOC_Os01g53930.1	OsHXK6	Os5	26017294	26022937	LOC_Os05g44760.1	OsHXK5
31	Os1	4820103	4823129	LOC_Os01g09460.1	OsHXK8	Os5	5337194	5341210	LOC_Os05g09500.1	OsHXK7
**Block**	**Oryza sativa gene location**			**Oryza sativa Gene ID**	**Oryza sativa Gene name**	**Medicago truncatula gene location**			**Medicago truncatula Gene ID**	**Medicago truncatula Gene name**
	**Chr**	**start**	**end**			**Chr**	**start**	**end**		
214	Os1	4820103	4823129	LOC_Os01g09460.1	OsHXK8	Mt8	43122065	43126983	Medtr8g102460.1	MtHXK3
**Block**	**Medicago truncatula gene location**			**Medicago truncatula Gene ID**	**Medicago truncatula Gene name**	**Medicago truncatula gene location**			**Medicago truncatula Gene ID**	**Medicago truncatula Gene name**
	**Chr**	**start**	**end**			**Chr**	**start**	**end**		
281	Mt6	33918506	33923707	Medtr6g088795.1	MtHXK2	Mt8	43122065	43126983	Medtr8g102460.1	MtHXK3
302	Mt8	43122065	43126983	Medtr8g102460.1	MtHXK3	Mt8	4574213	4578862	Medtr8g014530.1	MtHXK4
**Block**	**Phaseolus vulgaris gene location**			**Phaseolus vulgaris Gene ID**	**Phaseolus vulgaris Gene name**	**Phaseolus vulgaris gene location**			**Phaseolus vulgaris Gene ID**	**Phaseolus vulgaris Gene name**
	**Chr**	**start**	**end**			**Chr**	**start**	**end**		
106	Pv2	47615533	47622160	Phvul.002G308400.1	PvHXK1	Pv4	47830230	47834331	Phvul.004G175500.1	PvHXK2
164	Pv2	47615533	47622160	Phvul.002G308400.1	PvHXK1	Pv10	42638980	42644829	Phvul.010G144900.1	PvHXK4
167	Pv11	2110284	2115063	Phvul.011G023700.1	PvHXK3	Pv2	3391386	3395957	Phvul.002G034000.2	PvHXK5
**Block**	**Arabidopsis thaliana gene location**			**Arabidopsis thaliana Gene ID**	**Arabidopsis thaliana Gene name**	**Arabidopsis thaliana gene location**			**Arabidopsis thaliana Gene ID**	**Arabidopsis thaliana Gene name**
	**Chr**	**start**	**end**			**Chr**	**start**	**end**		
164	At4	14352036	14355103	AT4G29130.1	AtHXK1	At2	8570749	8574000	AT2G19860.1	AtHXK2
45	At5	18693683	18697659	AT1G50460.1	AtHXL1	At3	6994893	6998171	AT3G20040.1	AtHXL2

Table S4. The primers used in this study.

Primer Names	Sequence (5'-3')	Description
GmHXK1-qF	GAGCTTAGCTGGTGCAGGAA	RT-qPCR
GmHXK1-qR	TATAAGCCCCCATCGATGGC	RT-qPCR
GmHXK2-qF	GACTGGGGTGCCTTCTCAAA	RT-qPCR
GmHXK2-qR	CTGCGGCACACATTTTCCAA	RT-qPCR
GmHXK3-qF	TTACTGTTGCTGCCGACACT	RT-qPCR
GmHXK3-qR	TTGCTGAAGAAGGGGCAGAG	RT-qPCR
GmHXK4-qF	TTCGGGGCTGGCAAAATTTG	RT-qPCR
GmHXK4-qR	ACAGCAAAACCCTTGGTCCA	RT-qPCR
GmHXK5-qF	CAGTCCAATGGCACGCAAAA	RT-qPCR
GmHXK5-qR	CCTCCTTCCACAGTCACCAC	RT-qPCR
GmHXK6-qF	GCCGGAATCGTGGGGATAAT	RT-qPCR
GmHXK6-qR	TTGCCGAGCATTTCCCAGAT	RT-qPCR
GmHXK7-qF	GGAACTGGAGGAAGGGTGTG	RT-qPCR
GmHXK7-qR	TGTTCCTCTCTCGGTCCCAT	RT-qPCR
GmHXK8-qF	CAGGACAGAAGGGAGCTTGG	RT-qPCR
GmHXK8-qR	AGCAACCCGCATATCTAGGC	RT-qPCR
GmHXK9-qF	GGACTCCTATGATGGCTGCC	RT-qPCR
GmHXK9-qR	TCTGCGAGTCACCACATCAC	RT-qPCR
GmHXK10-qF	GGACTCCTATGATGGCTGCC	RT-qPCR
GmHXK10-qR	AGATACCAACAATGCCGGCA	RT-qPCR
GmHXK11-qF	AAAATGGCACGGTCCTTTGC	RT-qPCR
GmHXK11-qR	CCCAAGTACAAGCCAGACGT	RT-qPCR
GmHXK12-qF	ACCCTGAAGGAATTGCTGGG	RT-qPCR
GmHXK12-qR	ATTGGGAGTGAGAAGCTGCC	RT-qPCR
GmHXK13-qF	ACACAATAAAGGCCGGGGAG	RT-qPCR
GmHXK13-qR	AGAAGGGCTGCTCCAATTCC	RT-qPCR
GmHXK14-qF	AGATGCATGCTGGTCTTGCT	RT-qPCR
GmHXK14-qR	CCCTCCAAGGTCCAATGCAT	RT-qPCR
GmHXK15-qF	ATGTCAGCCATGCACCATGA	RT-qPCR
GmHXK15-qR	CCCAAAATACCAGCAGCAGC	RT-qPCR
GmHXK16-qF	GTTCGGAAGTTTGTGCCACC	RT-qPCR
GmHXK16-qR	GGAACTGGAGGAAGGGTGTG	RT-qPCR
GmHXK17-qF	GCAGACAAAGGGAACTGGGT	RT-qPCR
GmHXK17-qR	TGCAGACATGTCAGGTGTCC	RT-qPCR
GmHXK15-F	ATGGGGAAGGTCGCGGTG	cloning
GmHXK15-R	TTAAGACTCCTCCACTCCCAAATAT	cloning
